# Promoting Sustainable Mobility: A Perspective from Car and Public Transport Users

**DOI:** 10.3390/ijerph18094715

**Published:** 2021-04-28

**Authors:** Audrius Dėdelė, Auksė Miškinytė

**Affiliations:** Department of Environmental Sciences, Faculty of Natural Sciences, Vytautas Magnus University, 53361 Akademija, Lithuania; audrius.dedele@vdu.lt

**Keywords:** sustainable mobility, active travel mode, car user, public transport user, walking, cycling

## Abstract

Sustainable mobility is becoming a key factor in improving the quality of life of the residents and increasing physical activity (PA) levels. The current situation of sustainable mobility and its analysis is a first step in understanding the factors that would encourage residents to discover and choose alternative modes of travel. The present study examined the factors that encourage the choice of active modes of travel among urban adult population. Walking and cycling were analyzed as the most sustainable forms of urban mobility from the perspective of car and public transport (PT) users. Total of 902 subjects aged 18 years or older were analyzed in the study to assess commuting habits in Kaunas city, Lithuania. The majority (61.1%) of the respondents used a passenger vehicle, 28.2% used PT, and only 13.5% used active modes of travel. The results showed that safer pedestrian crossings, and comfortable paths were the most significant factors that encourage walking. A wider cycling network, and bicycle safety were the most important incentives for the promotion of cycling. Our findings show that the main factors encouraging walking and cycling among car and PT users are similar, however, the individual characteristics that determine the choice of these factors vary significantly.

## 1. Introduction

Sustainable or active travel modes have gained interest by researchers, general public, decision-makers, and planners as a potential solution to challenges facing in transportation, environmental, social, economic, and public health issues [1]. Active travel refers to walking, cycling, or using other form of physical activity (PA) for all types of journeys instead of using motorized transport.

In recent years, there has been an increased focus on the development of sustainable urban transport in European Union (EU) and because of this growing interest many strategic documents, projects, and initiatives were created at the EU level. One of the strategic documents is the sustainable urban mobility plan (SUMP) proposed by the European Commission (EC) as a policy tool that should contain a long-term and sustainable vision for cities in Europe to improve the overall quality of life for residents by addressing major challenges such as congestion, air and noise pollution, climate change, road accidents, unsightly on-street parking, and the integration of new mobility services [2]. The EC recommends that European towns and cities should develop SUMPs at the national level to improve the overall quality of life for residents and the urban environment. SUMPs give the highest priority to the most vulnerable group of road users-pedestrians, cyclists, and people with special needs. Kiba-Janiak and Witkowski [3] analyzed the formulation and implementation of SUMPs of 15 EU capital cities and found that those cities that have a comprehensive transport plan, have implemented the greatest number of measures of sustainable urban transport and have achieved the best results related to safety and emissions of air pollutants (NO_2_, PM). SUMP of Kaunas city has been recognized by the EC and received a special evaluation as one of the top three in the awards of sustainable urban mobility planning [4].

Previous studies have shown that individuals who walk or bike for commuting are more physically active and are more likely to meet public health recommendations [5]. The study in UK found that active travel was associated with relatively greater time spent in leisure moderate to vigorous PA and travel, and lower time in leisure sedentary screen time and sleep [6]. Higher levels of active travel were associated with higher levels of PA, lower risk of obesity, diabetes, CVD (cardiovascular disease), cancer, and all-cause mortality among adults [7,8,9]. There is evidence that regular PA can prevent against at least 25 chronic conditions and reduce the risk by 20–30% [10]. According to the WHO [11], in addition to health benefits of PA, the increase in active travel can also generate additional benefits, including cleaner air, less congested and safer roads, a reduced use of fossil fuels that are related to the goals of sustainable development. For all these reasons, it is important that local government authorities take some actions that could encourage greater use of alternative modes of transport.

However, planning and development of active travel infrastructures in cities faces numerous challenges and requires better understanding of both factors promoting active modes of travel as well as barriers to it. First, it is necessary to find out societal behavior and public attitudes toward sustainable modes of travel and factors that would encourage to use them. This study aimed to identify the main factors that would encourage car and public transport (PT) users to choose sustainable modes of travel more often. The main objective of this study was to identify active (walking and cycling) and passive (car and PT) travel mode users and to investigate factors that would encourage more frequent choice of walking and cycling from the perspective of car and PT users. The secondary objective was to determine how the choice of certain incentives depended on demographic and socioeconomic factors. It is hypothesized that individuals will choose different factors that promote walking and cycling depending on the use of a certain transport mode. Second, the study hypothesized that demographic and socioeconomic characteristics of car and PT users directly influence the choice of factors encouraging walking and cycling.

There is evidence that travel policies and interventions, transportation infrastructure, neighborhood patterns, social and psychological factors are associated with active travel modes. Findings from different studies confirm that quality of walking and cycling infrastructure as well as safety is one of the most important targets of active travel promotion policy and has a direct effect on the intention to use active modes of travel more [12,13,14]. A study in the United States investigated the associations between safety and security and travel modes. It was found that some measures of traffic safety (more comfortable facilities, sidewalks, traffic signals, etc.,) were positively associated with walking and cycling and a lack of personal security (higher levels of crime) were negatively associated with walking [15].

Previous studies have shown that comprehensive multi-level policies and strategies, new bike lanes may be effective in promoting active travel behavior changes [16,17]. Environmental factors related to walkability, areas with fewer physical barriers and better infrastructure are associated with more active travel [17,18]. A systematic review of walking and cycling promotion showed that the most effective interventions to promote active travel appeared to target accessibility and connectivity and traffic safety [19]. Several studies in the UK investigated the association between new and upgraded walking and cycling infrastructure and the change in use of it and demonstrated that new infrastructure can lead to an increase in active commuting [20] and a modal shift toward active travel [21].

Previous studies examining the main barriers to active commuting have shown that distance, comfort, safety, time spent on commuting, weather, cost, effort, and presence of bicycle parking are the most relevant discouraging factors [14,22]. The importance of these barriers differs by demographic and socioeconomic characteristics. There is evidence that a lack of safety among female and elderly is significantly a more important barrier to cycling and walking [14,23,24]. A study on frightening situations and their impacts on travel behavior among women in Austria showed that most incidents happen while women are walking (52.5%) and using PT (39%) [25]. A study in Brisbane, Australia, examined associations between crime and walking and found that high perceived crime was associated with reduced odds of transport walking, whereas high objective crime was associated with increased odds of transport walking [26].

Previous research on the association between active commuting and age are inconsistent. Some studies report that active travel declines with age and older adults are less likely to be active commuters [27,28]. However, travel patterns vary depending on the type of active mode. Older adults are more likely to walk than use a bike, although this is also influenced by different national bicycle cultures [29]. A lack of time was addressed as one of the most common barriers for active travel [16], especially among employed individuals [24]. A study of Canadian adults found that walking was more prevalent among the middle class, and unemployed individuals [24].

Our previous study on determinants of travel mode choice showed that the use of cycling was associated with gender, body mass index (BMI), travel distance, and travel cost. Age, BMI, and travel distance was the most significant factors for walking [29]. Previous findings have shown that the use of a car as the main mode of travel tends to increase among families with young children [30,31]. It is important to identify the main factors and individual characteristics that influence the choice of different travel modes to be able to target measures, campaigns, and initiatives along with behavioral changes toward more sustainable mobility, but it is equally important to identify factors encouraging walking and cycling among car and PT users.

The present study examined the factors that would encourage the use of active modes of travel among urban adult population in the city of Kaunas, Lithuania. This study contributes to the existing body of knowledge on the factors that promote sustainable mobility by analyzing walking and cycling separately from the perspective of car and PT users.

Travel behavior studies suggest that walking and cycling should be analyzed separately, because they are seen differently by people and policies to encourage these active travel modes should be different [32]. We investigated differences in factors encouraging walking and cycling between car and PT users and the influence of individual characteristics on the choice of these factors in order to assess how to contribute to the promotion of sustainable mobility in an urban Eastern Europe setting, changes in the improvement of infrastructure, which should ensure more convenient connections for all road users and the promotion of non-motorized transport and pedestrian mobility. However, promoting sustainable mobility requires not only a convenient infrastructure, but also a change in the habits of the population, which is a difficult process. In SUMPs, all measures are more focused on the improvement and development of infrastructure but changing people’s habits is also very important. Therefore, various campaigns, initiatives, and encouragement of target population groups to try another mode of travel would contribute to sustainable urban mobility.

## 2. Materials and Methods

### 2.1. Study Design and Study Population

A cross-sectional study on commuting habits of Kaunas residents was conducted in Kaunas city, Lithuania. Respondents were selected using the random sampling method based on gender and age in proportion to the size of the Kaunas population. The survey included permanent residents of Kaunas city who were 18 years of age or older. The participants were recruited and interviewed by telephone from September to November 2017. The survey was completely anonymous, and no identifiable personal details were collected from the respondents. The study received an approval from Kaunas Regional Biomedical Research Ethics Committee (Approval No. BE-2-16). Main outcome measure was active (walking and cycling) and motorized (car and PT) modes of travel and factors that encourage the use of active modes of travel. In total, 1111 participants completed the questionnaire. Among these participants, only those who indicated that they regularly use at least one of the analyzed modes of travel more than once a week were included in the present study. Individuals who choose a different mode of travel depending on the season were assigned to both groups. Thus, the data of 902 participants were analyzed in this study to identify the demographic, socioeconomic, and other characteristics of active travel users and to investigate the factors that would encourage walking and cycling among car and PT users.

### 2.2. Study Area

The city of Kaunas (54°56′ N, 24°51′ E; altitude 24–90 m) is the second-largest city of Lithuania with a population of 292,691 in 2017 and distributed over a land area of 157 km^2^.

Kaunas is situated at strategically convenient position in terms of intersection of the main Lithuanian roads and the national and international integration axes and railways. Kaunas is well integrated not only into the general Lithuanian transport system, but also into the international transport corridor network. The city of Kaunas has a well-developed air transport infrastructure and is one of the largest air freight hubs in the Baltic States. An international airport is situated in close vicinity (the distance between the airport and the city center is 14 km) [33].

The population density of Kaunas city reached 1864 inhabitants/km^2^ in 2017. Almost half of the city′s territory is built up and the density of the street network is 6.48 km/km^2^ [34]. According to the data of the Lithuanian Department of Statistics (Statistics Lithuania), the total length of the Kaunas city street network is 918 km, of which 745 km has a pavement and 587 km has an improved pavement. Over the past decades, the level of motorization grew rapidly and steadily in Kaunas. According to the data of Statistics Lithuania, in 2017, the number of passenger cars per 1000 inhabitants in Kaunas city was 409 vehicles. This number increased in 2018 and 2019, respectively, 422 and 432 vehicles per 1000 inhabitants. In comparison, the EU average increased from 558 passenger cars per 1000 inhabitants in 2017 to 569 in 2019 [35].

According to the database of enterprises of Kaunas, workplaces, schools, kindergartens, and shopping centers are distributed proportionally in the city.

PT network is characterized by a high frequency of services and a concentrated network of routes so that it can meet the needs of passengers. In recent years, Kaunas PT park has been renovated, more convenient payment for travel has been introduced and the quality of services has increased. The priority is to ensure a shorter journey time so that public transport in Kaunas is faster, priority public transport lanes and a more attractive and simpler new ticketing system are provided [36]. The city′s route network consists of 15 trolleybus and 49 bus routes [37]. The central part of the city has good public transport service, and peripheral parts have service approximately at every 30 min. The average duration of a ride by PT in Kaunas is 29 min [38].

Kaunas is actively expanding its infrastructure by building new bicycle paths and renovating old ones. The city is implementing projects aimed at expanding the bicycle transport infrastructure in order to create an integrated bicycle network system and promote the mobility of the population by non-motorized vehicles [39,40]. According to the data of Statistics Lithuania, in 2019, there was 110 km of bicycle paths in Kaunas.

The city is constantly investing in the reconstruction and the development of the road and cycling networks as well as in the renewal of the urban public transport infrastructure [41,42].

### 2.3. Independent Variables

The survey included questions about demographic (age, gender, marital status, children), socioeconomic (education level, employment status, occupational groups, income), health-related (BMI, smoking habits, chronic disease, PA, sedentary behavior (SB)) factors, travel behavior and the use of active and motorized modes of travel. Adults with one or more child under the age of 18 years (<18 y) were coded as yes and those adults who reported that they do not have a child or have one or more child over the age of 18 were coded as no. Education level was classified as low education (12 or fewer years), medium (non-university), and high education (university degree) level. According to the employment status, participants were classified into four groups: employed, students, retired, and unemployed individuals. Occupations were classified according to the International Standard Classification of Occupations (ISCO) and grouped into white-collar (ISCO-88 major occupational groups 1–5) and blue-collar (ISCO-88 major occupational groups 6–9) workers [43]. According to the collar type of the occupation, white-collar workers are those who perform professional, managerial, or administrative jobs, typically in an office or other administrative setting. Blue-collar workers include those who work in hard manual labor and in many other types of physical work. According to the Lithuanian Department of Statistics (Statistics Lithuania), in 2017, the average monthly disposable income per household was around € 1000. Thus, we used this value to classify the study participants into two income groups: (1) ≤ € 1000 and (2) > € 1000.

Body mass index (BMI) was calculated as the ratio of weight (kg) to height squared (m^2^). Participants were classified into two smoking categories: current smokers and non-smokers. The chronic disease variable was measured using the question that asked the respondents whether a doctor had ever diagnosed them with one or more chronic diseases. Level of PA (min/week) was assessed based on the Global Physical Activity Questionnaire developed by World Health Organization (WHO) and was divided into two groups according to the WHO physical activity recommendations: (1) less than 150 min/week and (2) equal to or more than 150 min/week. In the study, the participants were asked “How many hours per day on average did you spend sitting outside of work?” They were asked to estimate in total the number of hours per day they spent sitting on a weekday and a weekend day, not including the time spent sitting at work. The participants indicated whether the mode of travel they used was a work commute or a non-work travel and a categorical variable commute type was used for the analysis.

**Figure 1:**
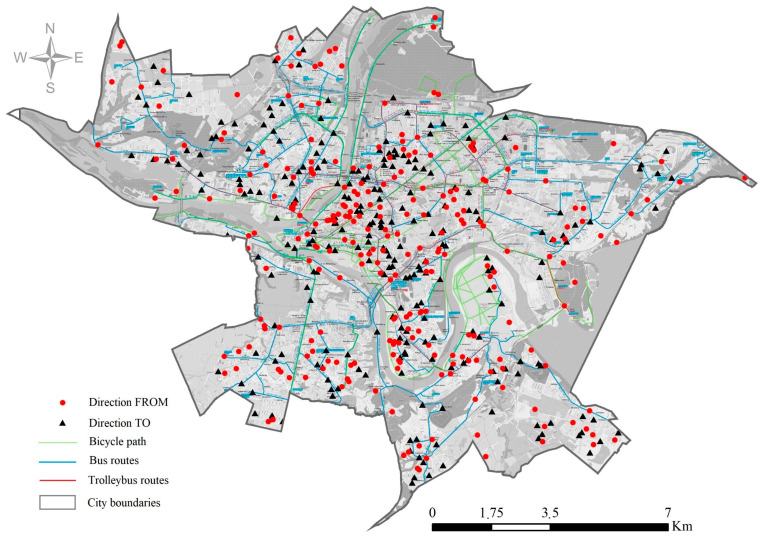
Kaunas city street, PT and bicycle path network and commuting patterns (origin and destination locations) of the study population.

Using the ArcGIS Network Analyst (Esri, Redlands, CA, USA), the length of the shortest route on the road and pedestrian/cyclist networks between two locations (home-work for employed individuals and home-other frequently visited location) was calculated. The shortest route was assessed for different modes of travel. To determine the optimal path along a linear network road and path networks of Kaunas city were used. Routes of cyclists were based on the combination of path and road networks. To find the shortest path from a starting location to a destination location (Figure 1), the Route Analysis tool from the Network Analyst toolbar was used. Details have been described in our previous study [29]. Once the travel distance for all participants was assessed, it was used as a variable for further analysis. Distance was used as a continuous and categorical variable. It was categorized into four groups: (1) <3 km, (2) 3–5 km, (3) 5–10 km, and (4) >10 km.

### 2.4. Outcome

According to the use of the travel mode, the participants were divided into active (bike users and walkers) and motorized (car and PT users) modes users. The participants were asked whether they walked, rode a bicycle, or used motorized transport (a car or PT) for daily commute to work or to some other frequently visited location. To identify factors that encourage more walking and cycling for daily travel, two questions were asked: “What would encourage you to walk more?” and “What would encourage you to cycle more?” The outcome variables were coded as dichotomous variables (1) if the travel mode and factor that encourages walking and cycling was chosen, and 0 if not.

### 2.5. Statistical Analysis

The descriptive statistics of the study population is expressed as the mean and standard deviation (SD) for continuous variables and frequencies and percentages for categorical variables. The independent t-test was used to compare the means of continuous variables by different travel modes. The chi-square test or Fisher’s exact test with less than 5 groups and its *p*-value was calculated for categorical variables to determine the associations between the use of active and motorized travel modes and individual characteristics of the study participants and factors that encourage walking and cycling among car and PT users separately. Multiple responses analysis was performed using the SPSS Multiple Response Sets function. The participants who selected more than one option for the following questions: “What would encourage you to walk more?” and “What would encourage you to cycle more?” were included in this analysis. Logistic regression analysis was used and odds ratios (ORs) with 95% confidence intervals (CIs) were calculated to assess the determinants of the choice of factors encouraging walking and cycling. We used binary logistic regression models to find associations between the choice of factors that encourage walking and cycling and individual characteristics of the study participants. In the multivariable logistic regression model, we adjusted for age, gender, marital status, health-related behaviors, and cardiometabolic risk factors. The *p*-value < 0.05 was used as the threshold of statistical significance. All statistical analyses were performed using SPSS version 25.0 (IBM Corp. released 2017. IBM SPSS Statistics for Windows, Version 25.0 Armonk, NY: IBM Corp). Data on travel distance were analyzed using ArcMap 10.4.1 (ESRI, ArcGIS Release 10.4.1. Redlands, CA, USA. Environmental Systems Research Institute).

## 3. Results

The demographic and socioeconomic characteristics of the study population are presented in Table 1 of the respondents, 55.3% were women. The average age of the respondents were 45.2 years. The majority (62.4%) of the respondents were married, had higher education (40.1%), and were employed (70.1%). The average BMI of the respondents was 25.9 kg/m^2^, and only 12.6% of the study population achieved recommended levels of physical activity. The majority (61.1%) of the respondents indicated using a car, 28.2% used PT, and 13.5% used active travel modes.

In order to investigate the factors that promote sustainable mobility, it is first important to understand the characteristics of people who walk or cycle or use motorized transport (Table 2). The results showed that older people (≥60 years), those who do not have minor children, have a lower level of education, are retired, have lower income, non-smokers, have lower BMI, do not have a chronic disease, achieve recommended levels of PA, spend more than 3 h a day sitting on weekday and weekend, travel the shortest distances (<3 km), and most for non-work purposes are more likely to choose active travel mode. The results demonstrated that there was a statistically significant difference in the average age, BMI, and travel distance between active and motorized travel users. Analyzing the differences in sedentary behavior between active and motorized travel users, we found that the average weekday and weekend day sitting time was higher among active travel users.

Factors encouraging people to choose the active mode of travel were analyzed for walking and cycling separately, from the perspective of car and PT users (Table 3). The results showed that safer pedestrian crossings (40.1% and 55.1%), and comfortable and high-quality paths for pedestrians (35.4% and 45.3%) would most encourage people to walk more, respectively among car and PT users. Analyzing the differences in car and PT users’ attitude toward factors influencing the decision to walk or bicycle, we found that comfortable and high-quality paths, safer pedestrian crossings, and crime prevention would encourage more PT users to walk (*p* < 0.05). Meanwhile, a shorter distance, and having more time would encourage more car users to walk. However, we did not find a statistically significant difference in a shorter distance between car and PT users. A higher percentage of PT users indicated that they walk enough compared to car users. Considering the determinants that would promote cycling, the results showed that a wider cycling network (40.5% and 34.6%), and bicycle safety (39.9% and 37.0%) would be the most important incentives for car and PT users to cycle more. Analyzing multiple responses, the results showed that a combination of factors such as comfortable and high-quality paths, safer pedestrian crossings and crime prevention would encourage to walk 11.6% and 16.1% of car and PT users, respectively, (*p* = 0.050). Meanwhile, comfortable, and high-quality paths, and safer pedestrian crossings would encourage to walk 22.7% and 35.0% of car and PT users, respectively. There was a statistically significant association between variables (*p* < 0.001). The results for cycling promotion showed that a set of factors such as a wider cycling network, more bike storage, bicycle safety, and theft prevention would encourage to cycle 12.5% of car and 8.7% of PT users (*p* = 0.066).

Demographic, socioeconomic, and other characteristics of the study participants have a significant impact on the choice of the factors that promote active travel modes. For multivariable logistic regression analysis, we selected the most influential factors that encouraged walking and cycling among car and PT users. The main factors that encouraged walking were chosen as follows: comfortable and high-quality paths, safer pedestrian crossings, crime prevention, a shorter distance, and having more time. The main factors that encouraged cycling were chosen as follows: a wider cycling network, more bike storage, bicycle safety, and theft prevention. After adjusting for potential confounding factors, the results showed that car users who chose comfortable and high-quality paths as a factor encouraging walking were more likely to be without minor children, unemployed, spend more time in sedentary behavior (SB) during weekday, and non-smokers (Table 4). Being older age, not having minor children, having higher education, spending more time in SB during weekday, having higher BMI, and being non-smoker were associated with more frequent choice of safer pedestrian crossings as a factor encouraging walking. Crime prevention was more important for women, those who spend more time in SB on weekends, and have higher BMI. A shorter distance and having more time would encourage more younger people, those who have minor children, and spend less time in SB both on weekdays and weekends to walk.

PT users, who would be encouraged to walk by comfortable and high-quality paths, tend to spend more time sitting on weekdays. Safer pedestrian crossings would encourage more unemployed people to walk. Crime prevention as a factor encouraging walking was more important for unemployed people, and those, who spend more time in SB on weekends. A shorter distance and having more time would encourage to walk those PT users who are employed and spend less time sitting on weekends.

Analysis of cycling promotion among car users showed that a wider cycling network would encourage younger people, those who spend more time in SB on weekends and have lower BMI to cycle more. These results were similar for PT users except that they tend to spend more time in SB on weekdays and there was no significant association with BMI (Table 5). More bike storage would encourage to cycle those car users who do not have minor children, have higher education, spend more time in SB on weekdays, have higher BMI, and non-smokers. Meanwhile, for PT users, being younger age was associated with more frequent choice of more bike storage. Bicycle safety was more important for those car users who have higher education, spend more time in SB on weekdays, have higher BMI, and are non-smokers. Meanwhile, among PT users, bicycle safety was more important for younger people and those who have lower education. Among car users, theft prevention to encourage cycling was more often chosen by people who have higher education, spend more time in SB on weekends and have higher BMI. Meanwhile among PT users, this factor was more frequently chosen by younger people, those who have lower education and spend less time in SB on weekdays.

## 4. Discussion

To promote sustainable mobility and to encourage people to cycle and walk more, it is important to increase the level of knowledge about the factors that would promote these modes of travel and identify the barriers that stop people from walking and cycling to their destinations. The principal aim of the present study was to identify the most important factors encouraging walking and cycling from the perspective of car and PT users and to determine the influence of individual characteristics on the choice of these encouraging factors.

Our research showed that safer pedestrian crossings was the most important factor that would promote walking both for car and PT users. Similar results in terms of road safety and active travel were found in previous studies. Panter and colleagues [44] in a study in Cambridge, UK found the association between greater perceived danger of cycling or of crossing the road and the increase in car trips. These results suggest that perceived safety and a safer infrastructure for pedestrians and cyclists could increase the choice of sustainable modes of travel.

The findings showed that factors associated with distance and time were important for car users to encourage walking. Previous studies have shown that one of the most important factors preventing people from choosing active modes of travel are distance, which is associated with increased travel time, and a lack of convenient infrastructure [45,46]. In addition, these results are also related to the individual characteristics of car users, because the car is mostly chosen by younger people, men, full-time workers, those having children and commuting longer distances, and they feel they have less time and opportunities to choose alternative modes of travel [29,45,47].

The results of the study demonstrated that factors encouraging cycling differed between car and PT users. The most significant factor for car and PT users was the wider cycling network and bicycle safety, respectively. There are greater safety and security concerns among PT users [48,49] and this may be one of the reasons why they are more concerned about bicycle safety compared to car users. Another reason may be related to the individual characteristics of PT users, as the majority of them are elderly, women, and children [50,51]. A study on encouraging and discouraging factors of cyclist from 20 different countries showed that the most common reasons discouraging the choice of the bicycle as a transport mode were a lack of safety and potential thefts, crash risk, long distances and topography, and a lack of proper infrastructure [52]. The study showed that having a bike would encourage more PT users to bicycle, meanwhile, having more time would encourage more car users to bicycle.

Our findings revealed that demographic and socioeconomic characteristics of car and PT users influenced their attitude toward factors encouraging walking and cycling. Having minor children, education level, SB, BMI, and smoking were the most significant factors influencing the choice of factors that would encourage walking among car users. Employment status, and SB were the most significant factors affecting the choice of factors that would encourage walking among PT users. Comfortable and high-quality paths would encourage to walk more unemployed people, travelling for non-work purposes, and those who spend more time in SB on weekdays and weekends. These results may be due to the fact that unemployed people have more free time and as our previous study has shown they are more physically active compared to employed individuals [53]. However, other studies show contrary results in terms of physical activity and employment status [54,55]. These inconsistent findings might be explained by the different population groups and their individual characteristics as the part of our population that is more prone to walking is older people, most of whom are retired and unemployed. Therefore, safer pedestrian crossings would encourage more older adults to walk. As implied by previous studies, elderly people are more concerned about the issues of safe mobility [56,57,58]. As expected, crime prevention as a factor encouraging walking was more important for women than for men. There is literature evidence that women tend to have a greater fear of crime than men, they feel more physically vulnerable, and are more concerned about their personal safety [59,60].

Among car users, safer pedestrian crossings and crime prevention as factors to encourage walking were more often chosen among highly educated people. Educational attainment is linked to lower crime rates [61]. Therefore, it can be argued that people with higher education pay greater attention to safety and crime prevention. Meanwhile, among PT users, safer pedestrian crossings and crime prevention were more often chosen by unemployed people, and those travelling more for non-work purposes. These results are related to the fact that older people, retirees, the unemployed, and those with lower incomes are more likely to use PT [31].

Among car users, younger people, those who have minor children, and spend less time in SB indicated that a shorter distance and having more time would encourage to walk more. Meanwhile among PT users, employed adults, those who commute to work, and spend less time in SB indicated that a shorter distance and having more time would encourage walking. These findings indicate that employed people and those who have minor children might have greater demands on their time and thus, prefer faster and more convenient modes of motorized transport [45,47].

The results of cycling promotion showed that the most significant individual characteristics influencing the choice of factors encouraging cycling were education level, SB, and BMI among car users and age, education level, and SB among PT users. Among car users, younger adults, those who spend more time in SB on weekends, and have lower BMI would be more encouraged to cycle by wider cycling network. Car users who have higher education, do not have minor children, spend more time sitting on weekdays, have higher BMI, and non-smokers indicated that the main factors encouraging cycling are more bike storage and bicycle safety. Theft prevention to encourage cycling was mostly chosen by highly educated people, those who tend to spend more time in SB on weekends and have higher BMI. Our findings show that highly educated people are more concerned about transport safety and thief prevention. Among PT users, younger people, those who have lower education, and spend less time in SB on weekdays would be encouraged to cycle more by analyzed factors. The findings of this study suggest that younger adults are more likely to cycle. Similar results were obtained in a study in the UK, which showed that cycling dominated among younger adults [62]. Demographic and socioeconomic factors play an important role in the choice of travel modes. Car and PT users are different in their demographic and socioeconomic characteristics and travel profiles. Therefore, individual characteristics associated with adult’s choice of encouraging factors differed significantly among car and PT users.

City government faces the challenge of encouraging residents to discover sustainable modes of travel such as walking and cycling. To encourage residents to choose active modes of travel, it is important to expand and improve infrastructure of cycling and walking routes that is adapted for more convenient and safer sustainable mobility of people in the city. It is important to build bicycle storage facilities in public spaces and near apartment buildings, to install electronic passenger information systems at bus stops and PT, because without the right network it is very difficult to achieve a positive impact in sustainable mobility of people. The solutions of Kaunas General Plan and Kaunas Sustainable Urban Mobility Plan should ensure the development of infrastructure. It is important that the planned development is integrated and sustainable.

## 5. Limitations

This study contains few limitations that could be enhanced in future studies. Although questionnaires are widely used in these types of studies [22,27] as the most accessible and commonly used method for studies of a large population, the behavioral factors of the study population and factors encouraging walking and cycling were self-reported. A self-report data collection method using formalized questionnaire may lead to a common source of biases. However, the survey was anonymous, and it is likely that this should have reduced the potential biases associated with making people feel uncomfortable or compromising answering personal or sensitive questions.

Although the sample size of our study was not small, having a larger sample size would have allowed a more detailed assessment of those encouraging factors that were less chosen. However, the sample size did not negate significance of our findings as the study population was representative to the entire population of Kaunas city.

The study used a cross-sectional design and, therefore, cannot be used to infer causality.

Our questionnaire did not include some other important barriers to active travel related to topography, weather, green spaces, and convenience. Future enhancements of these study components could be performed to strengthen the findings of the study.

## 6. Conclusions

The most important factors encouraging sustainable modes of travel were examined from the perspective of car and PT users. The findings showed that safer pedestrian crossing was the most important factor encouraging walking both for car and PT users. However, there were differences in car and PT users’ attitudes toward factors encouraging cycling. Our findings indicated that a wider cycling network is of primary importance to car users, while public transport users identified bicycle safety as the key factor.

The study showed that the main factors encouraging walking and cycling among car and PT users were similar, however, demographic, and socioeconomic characteristics that determine the choice of these factors vary significantly between car and PT users.

The study also suggests that future research could involve individuals who have already changed their mobility behavior toward more active travel and investigate the causes and factors that influenced these changes.

## Figures and Tables

**Table 1:** Characteristics of the study population (*n* = 902).

Variable	*n* (%)
Sex	
Women	499 (55.3)
Men	403 (44.7)
Age group, years	
≤39	362 (40.1)
40–59	357 (39.6)
≥60	183 (20.3)
Age (years), mean (SD)	45.4 (15.0)
Marital status	
Married	563 (62.4)
Divorced	103 (11.4)
Single	179 (19.8)
Widowed	57 (6.3)
Education level	
Low	294 (32.6)
Medium	246 (27.3)
High	362 (40.1)
Employment status	
Employed	632 (70.1)
Student	32 (3.5)
Retired	126 (14.0)
Unemployed	112 (12.4)
Occupational group (*n* = 579)	
White-collar	430 (74.3)
Blue-collar	149 (25.7)
Income (Eur) (*n* = 692)	
≤1000	378 (54.6)
>1000	314 (45.4)
BMI (kg/m^2^) (*n* = 719)	
<25	319 (44.4)
25–29	295 (41.0)
≥30	105 (14.6)
BMI, mean (SD)	25.9 (4.4)
Chronic disease	251 (27.8)
PA	
<150	788 (87.4)
≥150	114 (12.6)
Active travel mode users	122 (13.5)
PT users	254 (28.2)
Car users	551 (61.1)

SD: standard deviation; PA: physical activity; PT: public transport.

**Table 2:** Individual characteristics of the study participants by active and motorized travel modes (*n* = 902).

Variable	Active Travel Modes *n* (%)	Motorized Travel Modes *n* (%)	*p*-Value
Sex			0.348
Women	70 (14.0)	429 (86.0)	
Men	52 (12.9)	351 (87.1)	
Age group, years			<0.001 *
≤39	32 (8.8)	330 (91.2)	
40–59	28 (7.8)	329 (92.2)	
≥60	62 (33.9)	121 (66.1)	
Mean (SD)	54.1 (18.8)	44.1 (13.8)	<0.001 *
Marital status			<0.001 *
Married	65 (11.5)	498 (88.5)	
Divorced	14 (13.6)	89 (86.4)	
Single	21 (11.7)	158 (88.3)	
Widowed	22 (38.6)	35 (61.4)	
Minor children			<0.001 *
No	103 (17.5)	486 (82.5)	
Yes	19 (6.1)	294 (93.9)	
Education level			0.032 *
Low	52 (17.7)	242 (82.3)	
Medium	31 (12.6)	215 (87.4)	
High	39 (10.8)	323 (89.2)	
Employment status			<0.001 *
Employed	35 (5.5)	597 (94.5)	
Student	9 (28.1)	23 (71.9)	
Retired	52 (41.3)	74 (58.7)	
Unemployed	26 (23.2)	86 (76.8)	
Occupational group			0.065
White-collar	27 (6.3)	403 (93.7)	
Blue-collar	4 (2.7)	145 (97.3)	
Income, Eur			<0.001 *
≤1000	60 (15.9)	318 (84.1)	
>1000	20 (6.4)	294 (93.6)	
Smoking			0.002 *
No	97 (15.8)	518 (84.2)	
Yes	25 (8.7)	262 (91.3)	
BMI, kg/m^2^			0.153
<25	29 (9.1)	290 (90.9)	
25–29	19 (6.4)	276 (93.6)	
≥30	4 (3.8)	101 (96.2)	
Mean (SD)	24.8 (3.3)	26.0 (4.5)	0.014 *
Chronic disease			<0.001 *
No	68 (10.4)	583 (89.6)	
Yes	54 (21.5)	197 (78.5)	
PA, min/week			<0.001 *
<150	17 (2.2)	771 (97.8)	
≥150	105 (92.1)	9 (7.9)	
SB, hours			
Weekday, mean (SD)	3.4 (1.7)	2.8 (1.7)	0.001 *
Weekend day, mean (SD)	3.5 (1.7)	3.2 (1.7)	0.049 *
Distance, km			<0.001 *
<3	26 (17.4)	123 (82.6)	
3–5	19 (14.0)	117 (86.0)	
5–10	10 (4.3)	223 (95.7)	
>10	4 (1.6)	240 (98.4)	
Mean (SD)	3.8 (2.5)	6.9 (3.5)	
Commute type			<0.001 *
Work	35 (5.5)	597 (94.5)	
Non-work	87 (32.2)	183 (67.8)	

* Significant level ≤ 0.05. Categorical variables—Chi square test, continuous variables—the independent-samples t-test. SD: standard deviation; PA: physical activity; PT: public transport; SB: sedentary behavior.

**Table 3:** Factors that promote walking and cycling among car and PT users.

Factors	Car Users	PT Users	*p*-Value
Walking promotion			
Comfortable and high-quality paths	195 (35.4)	115 (45.3)	0.005 *
Safer pedestrian crossings	221 (40.1)	140 (55.1)	<0.001 *
Crime prevention	122 (22.1)	87 (34.3)	<0.001 *
Shorter distance	47 (8.5)	15 (5.9)	0.123
Have more time	46 (8.3)	11 (4.3)	0.024 *
Better weather	9 (1.6)	4 (1.6)	0.607
Better health	4 (0.7)	4 (1.6)	0.222
Walk enough	16 (2.9)	15 (5.9)	0.034
Cycling promotion			
Wider cycling network	223 (40.5)	88 (34.6)	0.066
More bike storage	161 (29.2)	76 (29.9)	0.451
Bicycle safety	220 (39.9)	94 (37.0)	0.239
Theft prevention	122 (22.1)	58 (22.8)	0.447
Have a bike	7 (1.3)	10 (3.9)	0.017 *
Shorter distance	9 (1.6)	1 (0.4)	0.125
Have more time	21 (3.8)	3 (1.2)	0.028 *
Better weather	5 (0.9)	1 (0.4)	0.387
Better health	4 (0.7)	5 (2.0)	0.118
Bike enough	10 (1.8)	7 (2.8)	0.268

* Significant level ≤ 0.05. Chi-square test, less than five groups—Fisher’s Exact Test. PT: public transport.

**Table 4:** Associations between individual characteristics of the study participants and factors encouraging walking among car and PT users.

Variable	Factors															
	Comfortable and High-Quality Paths				Safer Pedestrian Crossings				Crime Prevention				Shorter Distance and Having More Time			
	B	S.E.	OR	95% CI	B	S.E.	OR	95% CI	B	S.E.	OR	95% CI	B	S.E.	OR	95% CI
Car users																
Age	−0.01	0.01	0.99	0.98–1.01	0.03	0.01	1.03 **	1.01–1.04	0.001	0.01	1.00	0.98–1.02	−0.05	0.01	0.96 ***	0.93–0.98
Gender (ref. Male)	0.10	0.19	1.10	0.76–1.60	0.14	0.19	1.15	0.80–1.66	0.58	0.22	1.78 **	1.17–2.73	−0.06	0.25	0.95	0.58–1.55
Minor children (ref. No)	−0.53	0.22	0.59 *	0.38–0.91	−0.63	0.22	0.53 **	0.35–0.81	−0.04	0.25	0.96	0.59–1.56	0.63	0.28	1.88 *	1.08–3.26
Education (ref. Higher)	−0.36	0.22	0.70	0.46–1.07	−0.59	0.22	0.56 **	0.36–0.85	−0.46	0.27	0.63	0.37–1.07	0.30	0.27	1.35	0.80–2.30
Employment status (ref. Employed)	0.74	0.25	2.10 **	1.30–3.39	0.08	0.25	1.08	0.66–1.76	−0.01	0.29	0.99	0.56−1.75	−0.55	0.39	0.57	0.27−1.23
Weekday SB	0.10	0.05	1.10	0.99−1.23	0.15	0.06	1.17 **	1.05−1.30	−0.10	0.07	0.91	0.80−1.04	−0.51	0.11	0.60 ***	0.49−0.75
Weekend SB	0.20	0.06	1.22 ***	1.09−1.37	0.05	0.06	1.06	0.95−1.18	0.33	0.07	1.39 ***	1.22−1.58	−0.35	0.08	0.70 ***	0.60−0.83
BMI	−0.03	0.03	0.97	0.92−1.02	0.11	0.03	1.11 ***	1.05−1.18	0.07	0.03	1.07 *	1.01−1.14	−0.03	0.04	0.97	0.90−1.04
Smoking (ref. No)	−0.65	0.21	0.52 **	0.35−0.79	−0.78	0.21	0.46 ***	0.31−0.69	−0.35	0.25	0.71	0.44−1.15	0.15	0.27	1.16	0.69−1.95
PT users																
Age	−0.01	0.01	1.00	0.98−1.01	0.01	0.01	1.00	0.99–1.02	−0.004	0.01	1.00	0.98–1.01	−0.01	0.02	0.99	0.96–1.02
Gender (ref. Male)	0.52	0.37	1.68	0.81–3.47	0.51	0.37	1.67	0.82–3.40	0.68	0.42	1.98	0.87–4.53	0.84	0.68	2.32	0.62–8.77
Minor children (ref. No)	0.58	0.34	1.78	0.92–3.45	−0.22	0.34	0.80	0.41–1.56	−0.12	0.36	0.89	0.43–1.81	−0.22	0.52	0.80	0.29–2.22
Education (ref. Higher)	−0.10	0.27	0.91	0.54–1.52	0.44	0.27	1.55	0.91–2.64	0.25	0.28	1.29	0.74–2.23	0.38	0.44	1.46	0.62–3.45
Employment status (ref. Employed)	0.37	0.28	1.44	0.84–2.49	0.79	0.29	2.21 **	1.26–3.88	0.74	0.30	2.10 *	1.18–3.75	−1.53	0.58	0.22 **	0.07–0.68
Weekday SB	0.15	0.08	1.16 *	1.00–1.36	0.10	0.08	1.10	0.94–1.29	−0.13	0.09	0.88	0.74–1.05	−0.25	0.16	0.78	0.56–1.07
Weekend SB	0.06	0.08	1.07	0.93–1.24	0.05	0.08	1.05	0.90–1.22	0.36	0.09	1.43 ***	1.20–1.70	−0.30	0.15	0.74 *	0.55–0.99
BMI	−0.02	0.03	1.02	0.95–1.08	0.06	0.04	1.06	0.98–1.14	0.02	0.04	1.02	0.95–1.09	0.00	0.06	1.00	0.90–1.12
Smoking (ref. No)	−0.33	0.34	0.72	0.37–1.39	−0.62	0.34	0.54	0.28–1.05	−0.09	0.36	0.91	0.45–1.84	0.20	0.52	1.22	0.44–3.40

* *p* < 0.05; ** *p* < 0.01; *** *p* < 0.0001; adjusted for age, gender, marital status, education level, employment status. S.E.: standard error; SB: sedentary behavior; BMI: body mass index.

**Table 5:** Associations between individual characteristics of the study participants and factors encouraging cycling among car and PT users.

Variable	Factors															
	Wider Cycling Network				More Bike Storage				Bicycle Safety				Theft Prevention			
	B	S.E.	OR	95% CI	B	S.E.	OR	95% CI	B	S.E.	OR	95% CI	B	S.E.	OR	95% CI
Car users																
Age	−0.04	0.01	0.96 ***	0.95–0.98	0.01	0.01	1.00	0.99–1.02	−0.001	0.01	1.00	0.98–1.01	−0.002	0.01	1.00	0.98–1.02
Gender (ref. Male)	−0.05	0.19	0.96	0.66–1.38	−0.34	0.20	0.71	0.48–1.06	−0.12	0.19	0.89	0.62–1.28	0.20	0.22	1.23	0.80–1.88
Minor children (ref. No)	−0.22	0.21	0.81	0.53–1.22	−0.77	0.24	0.46 **	0.29–0.73	−0.36	0.21	0.70	0.46–1.05	−0.16	0.25	0.85	0.52–1.38
Education (ref. Higher)	−0.21	0.21	0.81	0.54–1.23	−0.66	0.24	0.52 **	0.32–0.83	−0.56	0.21	0.57 **	0.38–0.87	−0.72	0.28	0.49 *	0.28–0.84
Employment status (ref. Employed)	0.33	0.25	1.40	0.85–2.28	−0.14	0.28	0.87	0.51–1.50	−0.16	0.25	0.85	0.52–1.40	−0.36	0.32	0.70	0.38–1.30
Weekday SB	−0.06	0.06	0.95	0.85–1.05	0.11	0.06	1.12 *	1.00–1.25	0.14	0.05	1.15 *	1.03–1.27	−0.01	0.06	0.99	0.88–1.12
Weekend SB	0.16	0.06	1.18 **	1.06–1.32	0.04	0.06	1.04	0.93–1.17	0.08	0.06	1.08	0.97–1.21	0.28	0.07	1.32 ***	1.16–1.51
BMI	−0.06	0.03	0.94 *	0.89–0.99	0.08	0.03	1.08 **	1.02–1.14	0.09	0.03	1.09 **	1.03–1.15	0.08	0.03	1.09 **	1.02–1.15
Smoking (ref. No)	−0.04	0.20	0.96	0.65–1.42	−0.63	0.22	0.54 **	0.35–0.83	−0.70	0.21	0.50 **	0.33–0.74	−0.11	0.24	0.90	0.56–1.44
PT users																
Age	−0.03	0.01	0.97 ***	0.95–0.99	−0.03	0.01	0.98 **	0.96–0.99	−0.02	0.01	0.98 **	0.96–0.99	−0.03	0.01	0.97 **	0.95–0.99
Gender (ref. Male)	−0.20	0.37	0.82	0.39–1.70	−0.03	0.39	0.97	0.46–2.07	0.48	0.39	1.62	0.76–3.44	−0.05	0.42	0.95	0.42–2.18
Minor children (ref. No)	−0.03	0.35	0.97	0.49–1.91	−0.16	0.36	0.85	0.42–1.72	−0.61	0.35	0.55	0.27–1.09	−0.54	0.41	0.59	0.26–1.30
Education (ref. Higher)	0.18	0.28	1.19	0.69–2.07	0.38	0.29	1.46	0.83–2.57	0.69	0.28	1.99 *	1.16–3.43	0.89	0.32	2.43 **	1.30–4.53
Employment status (ref. Employed)	−0.03	0.30	0.97	0.54–1.74	−0.01	0.30	1.01	0.55–1.83	−0.26	0.29	0.77	0.44–1.38	0.24	0.33	1.27	0.66–2.44
Weekday SB	−0.18	0.09	0.84 *	0.70–0.99	0.03	0.08	1.03	0.88–1.22	−0.17	0.09	0.85	0.71–1.01	−0.24	0.11	0.79 *	0.63–0.98
Weekend SB	−0.08	0.09	0.93	0.79–1.10	0.02	0.08	1.02	0.86–1.20	0.000	0.08	1.00	0.86–1.17	0.16	0.09	1.17	0.98–1.40
BMI	−0.03	0.04	0.97	0.90–1.04	−0.002	0.04	1.00	0.93–1.07	0.04	0.04	1.04	0.97−1.12	0.01	0.04	1.01	0.94−1.09
Smoking (ref. No)	−0.32	0.35	0.73	0.37−1.45	−0.59	0.37	0.55	0.27−1.15	−0.30	0.35	0.74	0.38−1.47	−0.42	0.40	0.66	0.30–1.44

* *p* < 0.05; ** *p* < 0.01; *** *p* < 0.0001; adjusted for age, gender, marital status, education level, employment status. S.E.: standard error; SB: sedentary behavior; BMI: body mass index.

## Data Availability

The data presented in this study are available on request from the corresponding author.
